# What Medical Crowdfunding Campaigns Can Tell Us About Local Health System Gaps and Deficiencies: Exploratory Analysis of British Columbia, Canada

**DOI:** 10.2196/16982

**Published:** 2020-05-22

**Authors:** Jeremy Snyder, Marco Zenone, Valorie Crooks, Nadine Schuurman

**Affiliations:** 1 Faculty of Health Sciences Simon Fraser University Burnaby, BC Canada; 2 Department of Geography Simon Fraser University Burnaby, BC Canada

**Keywords:** crowdfunding, exploratory analysis, Canada, health system

## Abstract

**Background:**

There are a range of perceived gaps and shortcomings in the publicly funded Canadian health system. These include wait times for care, lack of public insurance coverage for dental care and pharmaceuticals, and difficulties accessing specialist care. Medical crowdfunding is a response to these gaps where individuals raise funds from their social networks to address health-related needs.

**Objective:**

This study aimed to investigate the potential of crowdfunding data to better understand what health-related needs individuals are using crowdfunding for, how these needs compare with the existing commentary on health system deficiencies, and the advantages and limitations of using crowdfunding campaigns to enhance or augment our understanding of perceived health system deficiencies.

**Methods:**

Crowdfunding campaigns were scraped from the GoFundMe website. These campaigns were then limited to those originating in the metropolitan Vancouver region of two health authorities during 2018. These campaigns were then further limited to those raising funds to allow the treatment of a medical problem or related to needs arising from ill health. These campaigns were then reviewed to identify the underlying health issue and motivation for pursuing crowdfunding.

**Results:**

We identified 423 campaigns for health-related needs. These campaigns requested CAD $8,715,806 (US $6,088,078) in funding and were pledged CAD $3,477,384 (US $2,428,987) from 27,773 donors. The most common underlying medical condition for campaign recipients was cancer, followed by traumatic injuries from collisions and brain injury and stroke. By far, the most common factor of motivation for crowdfunding was seeking financial support for wages lost because of illness (232/684, 33.9%). Some campaigns (65/684, 9.5%) sought help with purchasing medical equipment and supplies; 8.2% (56/684) sought to fund complementary, alternative, or unproven treatments including experimental interventions; 7.2% (49/684) sought financial support to cover travel-related costs, including in-province and out-of-province (49/684, 7.2%) travel; and 6.3% (43/684) campaigns sought help to pay for medication.

**Conclusions:**

This analysis demonstrates the potential of crowdfunding data to present timely and context-specific user-created insights into the perceived health-related financial needs of some Canadians. Although the literature on perceived limitations of the Canadian health system focuses on wait times for care and limited access to specialist services, among other issues, these campaigners were much more motivated by gaps in the wider social system such as costs related to unpaid time off work and travel to access care. Our findings demonstrate spatial differences in the underlying medical problems, motivations for crowdfunding, and success using crowdfunding that warrants additional attention. These differences may support established concerns that medical crowdfunding is most commonly used by individuals from relatively privileged socioeconomic backgrounds. We encourage the development of new resources to harness the power of crowdfunding data as a supplementary source of information for Canadian health system stakeholders.

## Introduction

### Background

A core aim of the Canadian health care system—and many other publicly funded health systems—is universal coverage for medically necessary services [1]. Nonetheless, Canadian health care users and policy makers report a range of perceived or experienced deficiencies in this system that impose financial costs on its users. These deficiencies include barriers to accessing care that motivate the use of private medical treatment and out-of-pocket costs when accessing publicly funded treatment [2,3].

Public reporting on systemic barriers to accessing care in Canada often focuses on wait times and health human resource shortages in key areas. A 2013 survey of Canadians found that waiting for a medical appointment and difficulty getting an appointment were the most common problems cited by those reporting access barriers. These barriers were greatest in relation to accessing specialist care, nonemergency surgery, and diagnostic testing. Canadians who were aged younger than 65 years, females, immigrants, people with some postsecondary education, and those with specific health needs were more likely to report barriers to accessing care than other groups. Geographically, Canadians in Quebec and the Western provinces, including British Columbia, were more likely to report access barriers than those living elsewhere in the country [4].

The Canadian Institute for Health Information has found that access barriers because of wait times vary by location and treatment sought. They report that 30% of Canadians perceive facing wait times for access to surgery for hip replacement or cataracts that are longer than recommended. At the same time, hip fracture repair was provided within the recommended wait times for 88% of Canadians, and 97% received timely radiation therapy [5]. British Columbia performs below the Canadian average in all wait time categories, with 67% of residents accessing hip replacement treatment within the recommended wait time, 59% for knee replacement, 85% for hip fracture repair, 64% for cataract surgery, and 93% for radiation therapy [6]. These perceived barriers to accessing care have been cited as factors motivating the expansion of private insurance in Canada and private payment for treatment domestically and abroad via medical tourism [7-9].

In addition to these perceived barriers to accessing care, several key gaps in coverage for services that are not covered under the Canada Health Act and thus are not part of the publicly funded health system have been identified [1]. These gaps include lack of or inadequate payment for prescription drugs, eye care, and dental care, which may be met by other social programs or private health insurance, depending on one’s income, place of residence, and employment [10,11]. Other nonreimbursed out-of-pocket expenses encountered by Canadians include parking payments at hospitals, medical devices used at home, accommodations while accessing nonlocal care, complementary and alternative treatments, home accessibility modifications, physical rehabilitation, in-home care, and travel costs [12-15]. Limited public coverage of prescription drugs, in particular, has received significant academic, policy, and political attention [16].

Some Canadians who are facing these perceived deficiencies in the Canadian health system are turning to medical crowdfunding to assist them with raising funds to support options such as seeking treatment abroad or paying for care and services that are not covered by the health and social care systems. Medical crowdfunding is a practice whereby individuals seek funds for health-related needs from their social networks via Web-based fundraising platforms. By far, the largest medical crowdfunding platform, GoFundMe, has been growing by 300% per year in 2016 and has raised funding from 25 million donors through 2 million crowdfunding campaigns [17]. In 2017, it was reported that it had raised US $3 billion since 2010 and has been raising US $140 million per month in donations. [18] More recently, the total raised by GoFundMe was reported as US $5 billion and growing [19].

### Objectives

Working from the premise that medical crowdfunding campaigns may provide unique user-focused insight into local health system gaps and deficiencies, in this exploratory analysis, we examine campaigns from two administrative regions of British Columbia. These administrative areas cover a range of communities, including dense urban centers, suburban regions, and less densely populated rural areas, all geographically contiguous and with access to extensive health system infrastructure. Our aim in undertaking this analysis was to better understand what health-related needs individuals in this area are crowdfunding for, how these needs compare with the existing commentary on health system deficiencies, and the advantages and limitations of using crowdfunding campaigns to enhance or augment our understanding of perceived health system deficiencies. We further aimed for this analysis to serve as a model for other analyses using crowdfunding data to enhance understanding of health-related needs.

## Methods

**Figure 1:**
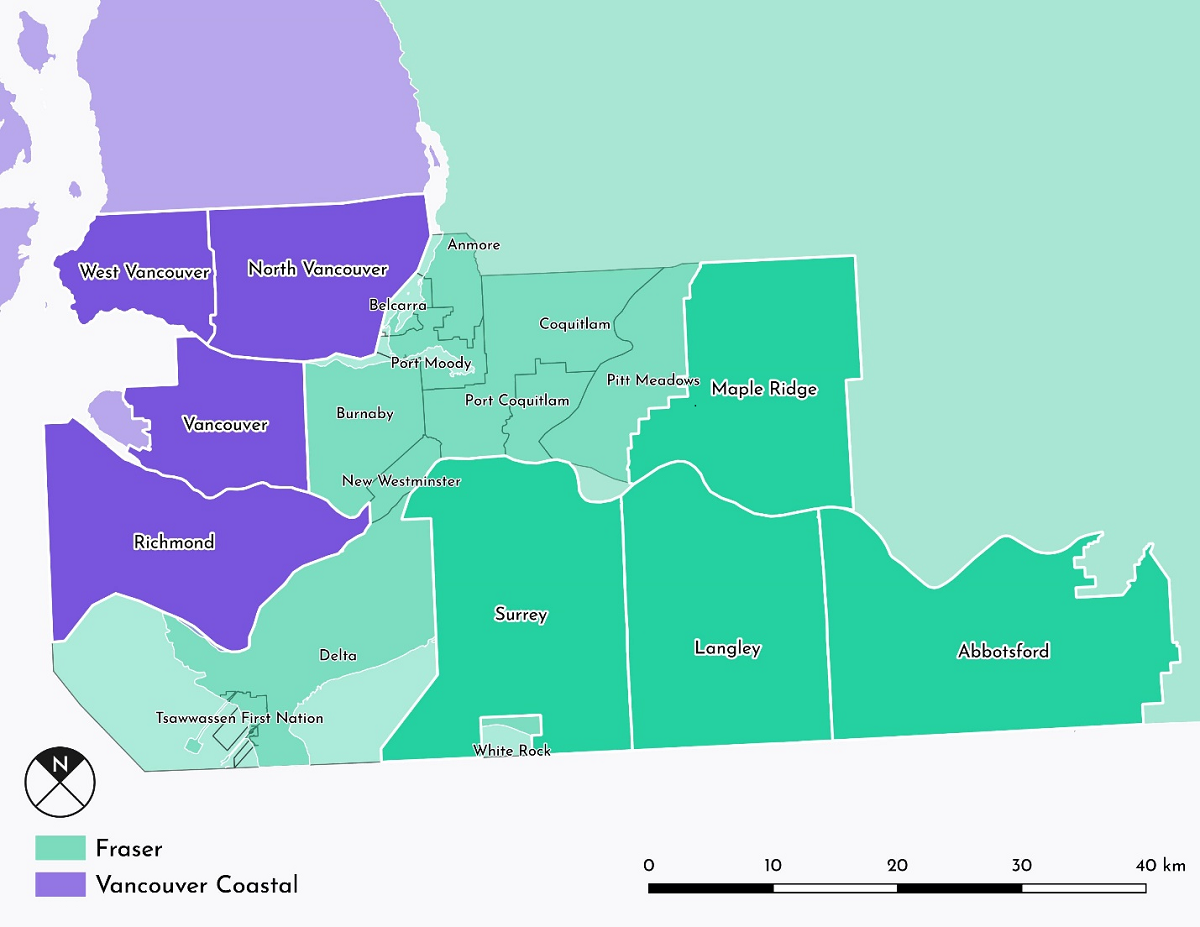
A map of campaigner locations.

We used an automated Web scraper to extract campaign data from GoFundMe.com. This scraper, the Crowdfunding for Health Research Portal, began recording data from GoFundMe crowdfunding campaigns in April 2019 using the GoFundMe.com sitemap. Information for every campaign listed on the sitemap was recorded at that time, including the title, text, updates, number of donors, money requested, money pledged, number of Facebook shares, and campaigner location. All campaigns listing locations in the Fraser Health Authority (FHA) region (n=2772) and metropolitan Vancouver portion of Vancouver Coastal Health Authority (VCHA) region (Vancouver, Richmond, North Vancouver, and West Vancouver; n=1971) were selected and recorded on a shared spreadsheet (see Figure 1). These regions are relatively densely populated geographically contiguous areas that are home to extensive health system infrastructure.

These campaigns were then restricted to fundraising categories, most likely to be health related (emergencies, family, medical, and uncategorized), and initiated in 2018.

After campaigns were extracted from the portal, the second author reviewed the campaigns, including those raising funds to allow treatment of a medical problem or related to needs arising from ill health. All authors independently reviewed 50 of the included campaigns and met to identify categories of health-related needs motivating crowdfunding, understood as needs triggered by, exacerbated by, or otherwise related to the underlying health condition. Included campaigns were then coded by JS and MZ for the requested health-related need, and the second author recorded the underlying health problem for each campaign. The fourth author then reviewed any codes flagged as uncertain by the first 2 reviewers and audited 5% of the coded campaigns to ensure consistency. Any disagreements were discussed and resolved by these reviewers. Given the exploratory purview of this analysis, sums were generated for the coded categories to allow for descriptive statistics to be generated.

## Results

We identified 423 campaigns for health-related needs between VCHA and FHA. These campaigns requested Can $8,715,806 (US $6,088,078) in funding and were pledged Can $3,477,384 (US $2,428,987) from 27,773 donors and shared 120,665 times on Facebook (see Table 1). In the FHA, campaigns were located most commonly in the cities of Surrey (47/275, 17.1%), Abbotsford (45/275, 16.4%), Langley (31/275, 11.3%), and Maple Ridge (26/275, 9.5%). In the VCHA, campaigns were divided between Vancouver (108/148, 73.0%), North Vancouver (27/148, 18.2%), Richmond (10/148, 6.8%), and West Vancouver (3/148, 2.0%; see Table 2). The most common underlying medical condition for campaign recipients was cancer, followed by traumatic injuries from collisions, and brain injury and stroke (see Table 3).

**Table 1:** Campaign engagement.

Category	Fraser Health Authority	Vancouver Coastal Health Authority	Total
Campaigns, n	275	148	423
Amount requested (Can $)	5,982,654 (US $4,178,944)	2,733,152	8,715,806‬
Money pledged (Can $)	1,939,932 (US $1,355,062)	1,537,452	3,477,384
Percentage pledged, %	32.4	56.3	39.9
Donors, n	15,826	11,947	27,773
Facebook shares, n	78,966	41,699	120,665

**Table 2:** Campaign location.

Location	Number of campaigns (N=423), n (%)	Population [20] (N=2,916,414), n (%)
Vancouver	108 (25.5)	672,963 (23.08)
Surrey	47 (11.1)	569,065 (19.51)
Abbotsford	45 (10.6)	151,923 (5.21)
Langley	31 (7.3)	154,867 (5.31)
North Vancouver	27 (6.4)	147,555 (5.06)
Maple Ridge	26 (6.1)	88,626 (3.00)
Burnaby	22 (5.2)	248,476 (8.52)
Chilliwack	21 (5.0)	90,931 (3.12)
Coquitlam	17 (4.0)	149,490 (5.16)
Port Coquitlam	17 (4.0)	62,844 (2.15)
New Westminster	12 (2.8)	76,799 (2.63)
Mission	10 (2.4)	41,503 (1.42)
Richmond	10 (2.4)	216,300 (7.42)
Delta	9 (2.1)	109,484 (3.75)
White Rock	6 (1.4)	21,370 (0.73)
Port Moody	4 (0.9)	35,613 (1.22)
Hope	3 (0.7)	6659 (0.23)
Pitt Meadows	3 (0.7)	19,772 (0.67)
West Vancouver	3 (0.7)	44,866 (1.54)
Agassiz	1 (0.2)	6624 (0.23)
Agassiz and Belcarra	1 (0.2)	684 (0.02)

**Table 3:** Underlying medical condition.

Medical condition	Fraser Health Authority (N=280), n (%)	Vancouver Coastal Health Authority (N=149), n (%)	Total (N=449), n (%)
Cancer	124 (44.3)	50 (33.6)	174 (40.6)
Trauma injuries	22 (7.9)	19 (12.8)	41 (9.6)
Brain injury/stroke	21 (7.5)	9 (6.0)	30 (7.0)
Unspecified/undiagnosed	22 (7.9)	6 (4.0)	28 (6.5)
Other	14 (5.0)	14 (9.4)	28 (6.5)
Heart disease/attack, diseases of circulatory system	14 (5.0)	4 (2.7)	18 (4.2)
Spinal disease/damage	8 (2.9)	7 (4.7)	15 (3.5)
Other neurological diseases	9 (3.2)	4 (2.7)	13 (3.0)
Kidney disease	8 (2.9)	3 (2.0)	11 (2.6)
Gastrointestinal disease/Crohn disease	5 (1.8)	6 (4.0)	11 (2.6)
Other genetic disorders	6 (2.1)	3 (2.0)	9 (2.1)
Diabetes	5 (1.8)	3 (2.0)	8 (1.9)
Gender affirmation	1 (0.4)	6 (4.0)	7 (1.6)
Amputation	3 (1.1)	4 (2.7)	7 (1.6)
Mental health and addiction treatment	2 (0.7)	5 (3.4)	7 (1.6)
Cerebral palsy/muscular dystrophy	5 (1.8)	1 (0.7)	6 (1.4)
Lyme disease	5 (1.8)	1 (0.7)	6 (1.4)
Burn injuries	5 (1.8)	0 (0.0)	5 (1.2)
Multiple sclerosis	1 (0.4)	4 (2.7)	5 (1.2)

After discussion, 13 categories of health-related needs motivating crowdfunding were identified. Campaigners were motivated by seeking funding for a variety of issues related to the recipient’s health needs (see Table 4). Of the 685 motivations recorded, including multiple motivations in several instances, by far, the most common was seeking financial support for wages lost because of illness (232/684, 33.9%). These included instances of income lost because of taking time off of paid employment because of the effects of illness, needing to take time off of paid employment for treatment, and needing to relocate for treatment. Such needs were often prompted by recipients reaching the limits in unemployment insurance coverage, as for the campaigner who wrote that “employment insurance does not cover much and only lasts for 10 weeks.”

**Table 4:** Motivation.

Motivation category	Fraser Health Authority (N=444), n (%)	Vancouver Coastal Health Authority (N=231), n (%)	Total (N=675), n (%)
Lost wages	153 (34.5)	79 (34.2)	232 (34.4)
Local travel expenses	35 (7.9)	14 (6.1)	49 (7.3)
Out-of-province travel	29 (6.5)	20 (8.7)	49 (7.3)
Medical equipment and supplies	43 (9.7)	22 (9.5)	65 (9.6)
Home accessibility improvements	16 (3.6)	3 (1.3)	19 (2.8)
Lack of travel insurance	0 (0.0)	2 (0.9)	2 (0.3)
Private care because of wait times	3 (0.7)	4 (1.7)	7 (1.0)
Private care because of quality of service	3 (0.7)	3 (1.3)	6 (0.9)
Medications	28 (6.3)	15 (6.5)	43 (6.4)
Caregiver expenses	11 (2.5)	1 (0.4)	12 (1.8)
Complementary, alternative, or unproven treatments	34 (7.7)	22 (9.5)	56 (8.3)
Elective or uninsured direct medical expenses	36 (8.1)	22 (9.5)	58 (8.6)
Unspecified or other direct medical costs	53 (11.9)	24 (10.4)	77 (11.4)

Other common motivations for crowdfunding for health-related expenses included elective or uninsured direct medical expenses (68/685, 9.9%). The treatments sought included diagnostic testing, fertility treatments, physiotherapy, and dental treatment, among others. Some campaigns (65/685, 9.5%) sought help with purchasing medical equipment and supplies. This equipment was typically intended for home use after discharge from hospital. Other campaigns (56/685, 8.2%) sought to fund complementary, alternative, or unproven treatments including experimental interventions. These requests ranged from complementary cancer treatments such as acupuncture to naturopathic treatment for Lyme disease and unproven stem cell interventions abroad. Seeking financial support to cover travel-related costs was another common motivation for crowdfunding, including in-province (49/685, 7.2%) and out-of-province (49/685, 7.2%) travel. Those traveling in-province typically needed help with gas expenses and parking for medical appointments or relocation to be closer to a medical center. Out-of-province travel included help relocating within Canada or, more commonly, travel abroad to privately purchase medical care. Help paying for medications was requested in 6.3% (43/685) campaigns. These were typically prescription medications, as in the case where “some of my medical issues require prescriptions that are not covered which means that I can't get them.” Unspecified health-related expenses were the second largest category (77/685, 11.2%). All other motivation categories were under 5% of all instances.

## Discussion

### Principal Findings

Our exploratory findings show the scope of crowdfunding in one metropolitan region in Canada. Despite universal public insurance coverage for Canadian residents, at least 423 individuals living in the areas covered by FHA and VCHA initiated crowdfunding campaigns in 2018, raising nearly Can $3.5 million (US $2,541,886). These campaigns impacted a wide network, as they received nearly 28,000 pledges of donations and were shared on Facebook over 120,000 times. Given that these campaigns may have been shared on other social media platforms and each share is viewed by multiple individuals, the impact of these campaigns is considerable.

As has been reported elsewhere, seeking funds to assist with addressing the costs of treating and managing cancer is by far the most common underlying health issue motivating crowdfunding by Canadians [21]. Other medical conditions linked to the crowdfunding campaigns reviewed in this analysis are less discussed in this literature, including trauma injuries because of collisions, brain injury and stroke, heart disease, and spinal injuries. This may reflect specific health needs in the region analyzed or broader crowdfunding trends across Canada.

Our findings demonstrate that out-of-pocket expenses incurred by people who were accessing care provided by the publicly funded health system were by far the most commonly cited reasons for using medical crowdfunding. Lost wages because of illness and accessing treatment was by far the most common motivation, appearing in 54.8% (232/423) campaigns. This may indicate insufficient access to or other limitations in employment benefits for residents. Indirect expenses such as in-province and out-of-province travel to access care (98/423, 23.2%), purchasing medical equipment and supplies (65/423, 15.4%), and making home accessibility improvements were also common (19/423, 4.5%). Although some people sought funds to cover the cost of privately-paid-for medical care, the overwhelming majority of campaigns was effectively illustrating the limits of the support available to those accessing the public system.

The extant literature on barriers to Canadians accessing medical care focuses on wait times for care. And there is some concern that these wait times drive Canadians to privately purchase care domestically or abroad. Meanwhile, this motivation was rarely found in the campaigns reviewed in this analysis, appearing in only 1.0% (7/100) instances. Similarly, accessing care privately because of the perceived (low) quality of care in the publicly funded system appeared only 0.9% (6/700) times. When the direct provision of care motivated crowdfunding, it was generally not to access private care more quickly but to afford elective or other uninsured forms of care (68/423, 16.1%), complementary or alternative care not covered by insurance (56/423, 13.2%), and medication not covered by insurance (43/423, 10.2%).

These findings demonstrate that campaigns exhibit spatial differences regarding crowdfunding requests between those living in the FHA and VCHA areas. Although both FHA and VCHA campaigns listed cancer as the most common underlying health condition motivating the campaign, FHA and VCHA contained 44.3% and 33.6% of cancer-related campaigns, respectively. Similarly, heart and circulatory diseases and kidney disease were much more common in FHA. Conversely, 7.9% of FHA campaigns involved accident trauma injuries compared with 12.3% for VCHA, and 4% of VCHA campaigns were for gender affirmation treatments compared with 0.4% for FHA. Motivations for engaging in crowdfunding were generally consistent across this region, although home accessibility improvements were nearly three times more common in FHA and private care because of wait times was more than twice as common in VCHA. Usage of crowdfunding varied greatly across metro Vancouver and the Fraser Valley as well, with higher usage rates compared with the underlying population for Abbotsford and Maple Ridge and lower usage rates for Richmond and Surrey. These differences merit continued exploration, as they could reflect spatially specific difficulties in meeting health-related needs.

The most striking difference between these two regions was in terms of success in meeting crowdfunding goals. FHA campaigns were pledged 32.4% of their requests compared with VCHA’s 56.3%. Evidence is emerging that crowdfunding campaigns may increase inequities by linking fundraising success to socioeconomic advantages and disadvantaging marginalized communities [22,23]. Average total annual income per person is Can $93,808 (US $65,417; Vancouver) and Can $83,850 (US $58,472; Richmond) in the VCHA vs Can $84,023 (US $58,593; Fraser East), Can $90,386 (US $63,031; Fraser North), and Can $97,301 (US $67,853; Fraser South) [24]. Statistics Canada distinguishes these two health authorities as falling into distinctly separate peer groups, with FHA having greater rates of visible minorities compared with VCHA (very high vs high) and Aboriginal residents (low vs very low) [25]. Life expectancies in VCHA and FHA are 84.4 and 82.8, respectively, and the infant mortality rates for VCHA and FHA are 3.0 and 3.3, respectively [26]. These socioeconomic and health inequality differences may be significant factors driving the differences in meeting campaign goals between those living in the FHA and VCHA regions. More generally, these findings raise questions as to whether the socioeconomic differences between regions are associated with differences in crowdfunding campaign success. However, this falls outside the scope of the current analysis and thus raises important implications for future research directions.

These findings can be used to support some existing arguments regarding the impact of insurance gaps and indirect medical expenses as pressing needs facing Canadians. Given current debates over medical prescription costs and PharmaCare expansion, this analysis demonstrates that such expenses are pushing some Canadians to turn to others for financial support to meet drug costs [27]. The prevalence of lost wages and travel expenses in these crowdfunding campaigns also show why indirect medical expenses must be part of the conversation of the social costs of ill health and gaps in social insurance. For example, this reflects public debates in British Columbia, where fees for parking costs at hospitals have been criticized as exploitative and unfair [28]. In total, 56 (13.2%) campaigns sought complementary and alternative medical interventions that, in some cases, are not evidence based, potentially risky to patients, and contain misinformation that is spread via these crowdfunding campaigns [29]. This finding can help inform debates about public funding for complementary and alternative treatments and confusion about their relationship to traditional, evidence-based treatment [30].

It is notable that some underlying health needs were largely not represented in these campaigns despite a prominent place in local discussion of the health system. Much recent discussion has taken place in metropolitan Vancouver around the opioid crisis, mental health and addiction issues, and the lack of adequate access to treatment to address these health needs [31]. However, these conditions were represented in only 1.6% (7/438) of the campaigns reviewed. This suggests that we should use campaigns to complement ongoing local dialog about health care but not replace other sources. Moreover, some of the most pressing health system priorities are a clear reflection of the socioeconomic gradient in communities. Noting that people were not crowdfunding for this care may add support to the literature that suggests that those who crowdfund are more educated and have higher socioeconomic status than the community at large.

### Limitations

This analysis has two main limitations, given the data used. First, crowdfunding data are self-reported and may be inaccurate. For example, the location of the campaign reflects the location of the campaign maker, who may be different from and in a different location than the campaign recipient. Campaigns were carefully reviewed to exclude cases where the recipient was clearly outside of the geographic area of interest. Second, because crowdfunding campaigns can be deleted, and data were collected in early 2019, many campaigns from the VHA and VCHA regions were likely not included in our findings. Thus, the findings here likely represent an undercount of the total crowdfunding activity in the FHA and VCHA.

### Conclusions

This analysis demonstrates the potential of crowdfunding data to present timely and context-specific user-created insights into the perceived health-related financial needs of some Canadians. Such insights can complement those generated by other sources, such as administrative and census data and even media and public discussions about health system reforms. New crowdfunding campaigns are being generated on an ongoing basis campaign-based data can be used to identify trends in perceived health-related financial needs as they develop as well as service gaps. These data are also context specific and thus can help to inform policy makers, patient advocates, health workers, and other stakeholders’ perceived health system deficiencies that lead to financial burdens for residents of Canada. Importantly, not all of the health-related needs described in these campaigns should be interpreted as identifying health and social system gaps that must be addressed. That said, a better understanding of trends around practices that fall well outside the scope of the Canada Health Act, such as accessing unproven medical interventions and traveling abroad for care, are also useful for health system stakeholders. This is because practices such as these have been shown to potentially introduce health system burdens and create ethical challenges for physicians [32].

We encourage the development of new resources to harness the power of crowdfunding data as a supplementary source of information for Canadian health system stakeholders. Although the findings of this study demonstrate the potential of these data to shed additional light on perceived health system deficiencies, they also show that context is of great importance in using these data. Despite their geographic proximity, the crowdfunding campaigns from these two health regions showed noteworthy differences between the percentages of requested funding that received. This finding lends support to existing questions about what factors determine crowdfunding success and whether this reflects larger social inequities. Finally, more research on crowdfunding campaigns across Canada, including those in rural settings, will allow regional differences in crowdfunding motivations and success to be identified.

## Abbreviations

FHA: Fraser Health Authority
VCHA: Vancouver Coastal Health Authority
